# Optimized assessment of both left and right ventricular parameters from multi-slice acquisitions of single orientations using reconstructed 3D cine data

**DOI:** 10.1186/1532-429X-14-S1-P289

**Published:** 2012-02-01

**Authors:** Daniel Messroghli, Markus Huellebrand, Darach O h-Ici, Anja Hennemuth, Titus Kuehne

**Affiliations:** 1 Congenital Heart Disease and Pediatric Cardiology, Deutsches Herzzentrum Berlin, Berlin, Germany; 2 Institute for Medical Image Computing, Fraunhofer Mevis, Bremen, Germany

## Background

The assessment of global left-ventricular (LV) parameters from a stack of multiple slices is usually performed in short-axis orientation, whereas transversal orientation is commonly preferred for the assessment of right-ventricular (RV) parameters. Other choices carry the risk of inaccuracies due to hindered delineation of valvular planes. The purpose of our study was to reconstruct 3D cine data sets in short-axis orientation from transversal image data and vice versa, and to assess the accuracy of global LV and RV parameters derived from these reconstructed data sets as compared to that from data sets with targeted acquisition in both orientations.

## Methods

In 10 patients with congenital heart disease (age 16 to 46 years, 4 male), multi-slice cine data sets encompassing both ventricles were acquired in both short-axis and transversal orientation (balanced steady-state free precession, 30 phases, slice thickness 6 mm, no inter-slice gap). LV and RV end-diastolic volumes (EDV), end-systolic volumes (ESV), and ejection fraction (EF) were derived from the acquired data sets. Using a custom-built CMR analysis program (CAIPI), transversal data sets were reconstructed from images acquired in short-axis orientation, and short-axis data sets were reconstructed from images acquired in transversal orientation. The same biventricular parameters were then derived from the reconstructed data sets using a semi-automatic contour detection tool from CAIPI.

## Results

On the acquired data sets, mean differences between ventricular parameters as assessed from short-axis and transversal orientation ranged from 0.4+/-10.4% (RV EF) to 12.3+/-9.7% (RV ESV). Bland-Altman comparison showed better agreement between LV parameters derived from reconstructed short-axis and acquired short-axis data sets than between acquired transversal and acquired short-axis data sets. In concordance, there was higher agreement between RV parameters derived from reconstructed transversal and acquired transversal data sets than between acquired short-axis and transversal data sets.

## Conclusions

The use of reconstructed 3D cine data sets allows for using optimal analysis planes for both ventricles with high diagnostic accuracy and without the need for time-consuming acquisition of two separate cine data sets.

## Funding

DFG, BMBF.

